# Alterations in mouse visceral adipose tissue mRNA expression of islet G‐protein‐coupled receptor ligands in obesity

**DOI:** 10.1111/dme.14978

**Published:** 2022-10-26

**Authors:** Tanyel Ashik, Vivian Lee, Patricio Atanes, Shanta J. Persaud

**Affiliations:** ^1^ Department of Diabetes, School of Cardiovascular and Metabolic Medicine & Sciences King's College London London UK

**Keywords:** adipose tissue, diabetes, G‐protein‐coupled receptors, ligands, mRNAs, obesity

## Abstract

**Background:**

Adipose tissue mass expansion in obesity leads to alterations in expression and secretion of adipokines, some of which may alter islet function by binding to G‐protein‐coupled receptors (GPCRs) expressed by islets. We have therefore quantified expression of mRNAs encoding islet GPCR ligands in visceral adipose tissue retrieved from lean and diet‐induced obese mice to determine alterations in islet GPCR ligand mRNAs in obesity.

**Methods:**

Epididymal adipose tissue was retrieved from C57BL/6 mice that had been maintained on a control‐fat diet (10% fat) or high‐fat diet (60% fat) for 16 weeks and RT‐qPCR was used to quantify mRNAs encoding ligands for islet GPCRs.

**Results:**

Of the 155 genes that encode ligands for islet GPCRs, 45 and 40 were expressed in visceral adipose tissue retrieved from lean and obese mice respectively. The remaining mRNAs were either expressed at trace level (0.0001% to 0.001% relative to *Actb* expression) or absent (<0.0001%). Obesity was associated with significant alterations in GPCR ligand mRNA expression in visceral adipose tissue, some of which encode for peptides with established effects on islet function (e.g. neuropeptide Y), or for GPCR ligands that have not previously been investigated for their effects on islets (e.g. (C‐C motif) ligand 4; Ccl4).

**Conclusion:**

Mouse visceral adipose tissue showed significant alterations in expression of mRNAs encoding islet GPCR ligands in obesity. Our data point to ligands of interest for future research on adipose‐islet crosstalk via secreted ligands acting at islet GPCRs. Such research may identify islet GPCRs with therapeutic potential for T2D.

**What's new?**

- Expansion of adipose tissue in obesity impairs metabolic homeostasis and can lead to T2D. There is only limited information available on adipose tissue‐islet crosstalk via secreted adipokines binding to islet G‐protein‐coupled receptors (GPCRs).
- We have quantified mRNAs encoding islet GPCR ligands in adipose tissue from lean and diet‐induced obese mice and found altered expression in obesity. Upregulation of adipokine mRNAs in obesity, such as that encoding chemokine (C‐C motif) ligand 4 (Ccl4), could have a detrimetal impact on islet function through inhibition of insulin release.
- Our data profiling adipose tissue‐derived islet GPCR ligand mRNAs may identify islet GPCRs with therapeutic potential for T2D.

## 1 INTRODUCTION

Approximately ~90% of patients diagnosed with T2D are either overweight or obese, and the risk of diabetes development is correlated not only with the presence but also the duration of excess adiposity.^1^ This establishes a simple but definitive link between adiposity and a state of defective glycaemic control, and the role of adipose tissue as a vital endocrine organ contributing to metabolic homeostasis remains at the heart of the T2D pathological process.

The progression of T2D reflects a slow decline in islet β‐cell function and an inability to secrete sufficient insulin in response to peripheral insulin resistance. The resulting chronic hyperglycaemia fuels the development of severe microvascular and macrovascular complications, so glycaemic control is a primary focal point for T2D treatment and management. Most current pharmacotherapies either increase insulin secretion or enhance insulin action to maintain plasma glucose within the desirable range. However, despite the wide range of diabetes medications available, a considerable proportion of patients still fail to achieve adequate glycaemic control^2^ so there is a considerable need to develop other, more efficacious pharmacotherapies for T2D.

Adipocytes are highly active sources of secretory products that are expressed and released in response to various hormonal and central afferent signals.^3^ Adipocyte‐derived secretory products include growth factors, extracellular matrix proteins, factors associated with lipid metabolism and secreted proteins known as ‘adipokines’.^4^ The diversity of the adipocyte secretome gives adipose tissue the ability to communicate with distinct organs to facilitate coordination of various physiological processes, including those concerning immunity, reproduction, neuroendocrine function and energy homeostasis.^3^

Leptin is a well‐characterised adipokine that acts centrally in the regulation of satiety, energy intake and the onset of puberty. Direct peripheral actions of leptin on rodent and human islet β‐cells have also been established, with reports of inhibitory effects on glucose‐stimulated insulin secretion in vitro and elevated plasma glucose levels in vivo.^5^, ^6^ Other adipocyte‐specific factors that are relevant to glucose homeostasis include resistin and adiponectin.^3^ These peptides have opposing effects on insulin‐stimulated glucose uptake and insulin sensitivity. Thus, resistin administration induces insulin resistance^7^ while high plasma levels of adiponectin are associated with improved insulin sensitivity.^8^ In addition, exogenous adiponectin acts directly at islets in vitro to potentiate glucose‐stimulated insulin secretion.^9^

Despite the longstanding knowledge that the adipokines leptin and adiponectin are key regulators of metabolic competence, there is little information available about whether adipokines can establish metabolic crosstalk between adipocytes and β‐cells via binding to β‐cell G‐protein‐coupled receptors (GPCRs). Thus, there are likely to be additional secreted adipokines, some of which act as GPCR ligands, which could be pharmacologically relevant in the treatment of T2D. For example, adipocytes secrete adipokines such as adipsin which stimulates insulin secretion in vitro and in vivo.^10^ Adipsin has been highlighted as an adipocyte‐derived peptide that protects β‐cells through increasing generation of the complement peptide C3a^11^ and we have previously reported that C3a has direct beneficial effects on islets through activation of the GPCR C3aR.^12^ The recent observation that diabetes risk is reduced with higher levels of circulating adipsin points to adipocyte‐derived peptides having therapeutic potential in T2D through β‐cell GPCR activation.^11^ While it is clear that adipsin is an interesting candidate for a novel T2D therapy, there are likely to be other, as yet unexplored, adipocyte‐derived GPCR‐activating peptides. These could act at islet GPCRs to improve insulin secretion and protect against β‐cell loss and/or promote β‐cell proliferation.

Another layer of complexity arises from observations that changes in mRNA expression of adipocyte‐derived peptides occur with alterations in metabolic status. Thus, exposure to an obesogenic or diabetic milieu alters adipocyte physiology^13^ and leads to an increase in the production of proinflammatory cytokines, such an IL‐6 and TNF‐α, that negatively impact insulin secretion through non‐GPCR signalling.^4^ This lends the possibility of exploiting the adipocyte secretome to identify islet GPCR targets with potential for T2D therapy. We have previously identified mRNAs encoding 293 GPCRs in human islets, but effects of a majority of the identified GPCRs on islet function remain unknown.^14^ Moreover, the expression profile of GPCR ligands in visceral adipocyte tissue has not yet been characterised, nor is it known whether they show altered expression in obesity. In this study, we have therefore retrieved visceral adipocyte tissue from mice fed control‐fat diet, or those that had been maintained on a high‐fat diet for 16 weeks to induce obesity, and quantified expression of mRNAs encoding ligands that can bind to islet GPCRs.

## 2 METHODS

### 2.1 Materials

Culture media and supplements, collagenase type I, TRIzol™ reagent and cDNA reverse transcription kit were supplied by Thermo Fisher Scientific. RNase‐free DNase set, miRNeasy Mini Kit, QuantiTect primers and QuantiFast SYBR Green PCR Kit were supplied by Qiagen Ltd. Adipose tissue homogenisation was achieved using a TissueLyser II and stainless‐steel beads also supplied by Qiagen Ltd. Reverse transcription was performed in a T100 thermal cycler (Bio‐Rad) and real‐time PCR was performed with a LightCycler® 480 (Roche Diagnostics). The concentration and purity of RNA samples were measured using a Nanodrop™ 1000 spectrophotometer (Thermo Fisher Scientific).

### 2.2 Mice

Experiments were performed in accordance with UK legislation under the Animals (Scientific Procedures) Act 1986 Amendment Regulations. Ten 8‐week‐old C57BL/6 male mice had ad libitum access to water and assigned to either control‐fat diet (10% fat) or high‐fat diet (60% fat) for 16 weeks prior to culling at 24 weeks of age. All animals were housed at 21–25°C with a 12‐h light–dark cycle.

### 2.3 RNA isolation from adipose tissue and qPCR analysis

The high triglyceride content and relatively low RNA content of adipocytes mean that standard RNA extraction protocols often lead to generation of low‐quality RNA, which can cause unreliable results. We therefore used a modification of previous protocols^15^, ^16^ to isolate RNA from mouse epididymal adipose tissue. In our protocol, both epididymal fat pads were retrieved from the abdominal cavity and processed together as a single sample. They were washed with KRBH buffer containing 100 U/ml penicillin and 100 mg/ml streptomycin and immediately snap‐frozen in liquid nitrogen. For processing, samples were placed on dry ice and, using a scalpel and forceps, approximately 100 mg of adipose tissue from each sample was retrieved and transferred to an Eppendorf tube. 1 ml of TRIzol® and stainless‐steel beads were added to the samples, which were homogenised using a Qiagen TissueLyser II (20 Hz, 2 min). Following homogenisation, samples were centrifuged (9500 *g*, 4°C, 30 min), the red phenol phase containing RNA was transferred to a new Eppendorf tube, TRIzol® was added to the RNA‐containing phenol phase to give a final volume of 1 ml and samples were vigorously shaken following the addition of 200 μl of chloroform. Samples were centrifuged (12,000 *g*, 4°C, 15 min) and approximately 600 μl of the upper aqueous phase containing RNA was transferred to new Eppendorf tubes, ensuring that 3–4 mm of the aqueous phase was left above the interphase to minimise the carryover of contaminating DNA. Subsequent steps utilised the Qiagen miRNeasy Mini Kit following the manufacturer's instructions. The concentration and purity of adipose tissue‐derived RNA samples were determined using a Nanodrop 1000 spectrophotometer and 400 ng of each RNA sample was reverse transcribed to cDNA. A Qiagen SYBR Green‐based qPCR protocol using QuantiTect primers (Table S1) was used to detect 155 genes encoding ligands targeting the 110 protein‐activated GPCRs that we have previously identified in islets,^14^ and these have been termed ‘islet GPCR ligands’ in this paper. *Lep* and *AdipoQ*, encoding leptin and adiponectin, respectively, were chosen as positive control genes and all mRNA levels were normalised to *Actb* levels in the same samples.

### 2.4 Data analysis

Gene expression relative to *Actb* was calculated using the ΔΔCt method (Pfaffl, 2001), where E = primer efficiency value; gioCt = Ct value of the gene of interest; hCt = Ct value of the housekeeping gene. Data analysis was performed on GraphPad Prism 8.4.0. Differences in mRNA expression between lean and obese groups were assessed with unpaired two‐tailed *t*‐tests. *p* < 0.05 was considered significant.

## 3 RESULTS

### 3.1 Mouse characteristics and expression of islet GPCR ligand mRNAs in visceral fat

**FIGURE 1:**
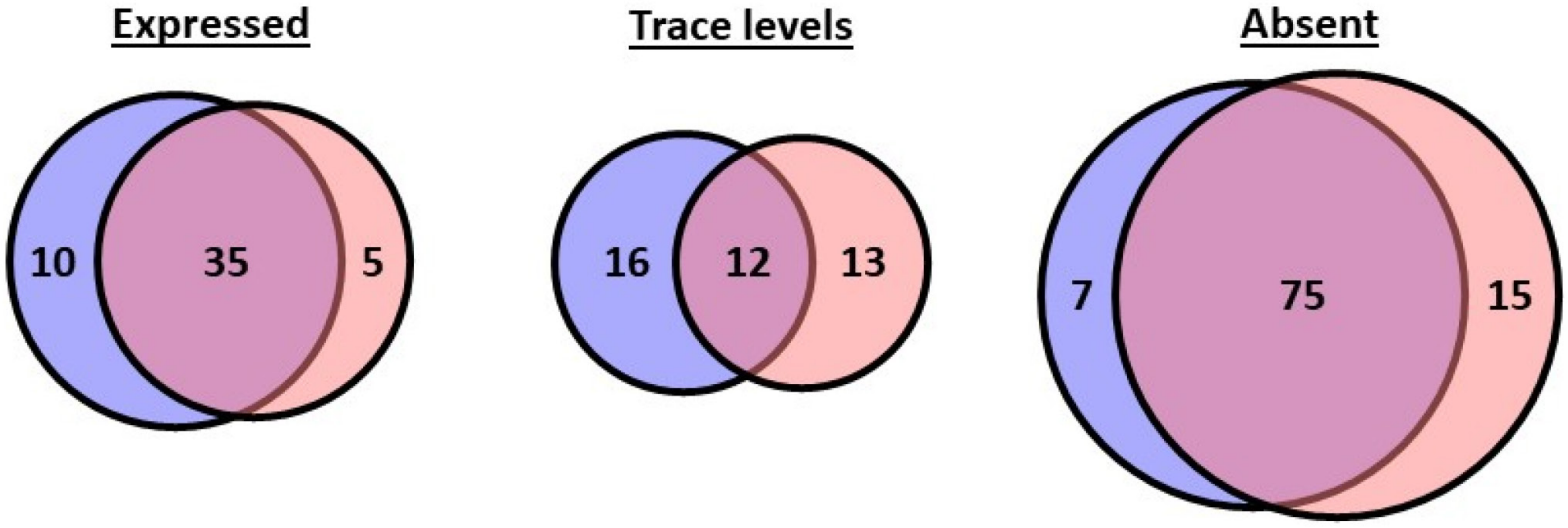
Proportions of islet GPCR ligand mRNAs classified as being expressed (>0.001% relative to *Actb*), at trace level (0.001 to 0.0001% relative to *Actb*) or absent (<0.0001% relative to *Actb* in visceral adipose tissue retrieved from lean and obese mice. Data were obtained by RT‐qPCR quantifications of RNA isolated from 5 lean (blue) and 5 obese (orange) mice. Genes common to both lean and obese mice are shown by the pink intersections.

The five mice fed on the 10% fat diet had a mean weight of 29.8 ± 0.15 g at the time of tissue retrieval, while those maintained on the 60% fat diet weighed 51.1 ± 0.59 g (*p* < 0.0001) and the fasting blood glucose levels were 5.3 ± 0.77 mM and 8.3 ± 0.47 mM (*p* < 0.05) respectively. RT‐qPCR indicated that of the 155 genes that encode ligands for islet GPCRs, 45 and 40 were expressed in visceral adipose tissue retrieved from control‐diet fed (lean) and high‐fat diet fed (obese) mice respectively (Figure 1). The remaining mRNAs were either expressed at trace level (0.0001% to 0.001% relative to *Actb* expression) or absent (<0.0001%). The Venn diagrams in Figure 1 show these three categories of GPCR ligand mRNA expression in adipose tissue from lean (blue) and obese (orange) mice and the overlap in expression of mRNAs is shown in the pink intersection. Thus, 35 of the expressed genes were common to the adipose tissue from both lean and obese mice. In addition, of the 155 mRNAs quantified 28 and 82 were expressed at trace levels or absent or in lean mice, while 25 and 90 were trace or absent in obese mice, with overlaps of 12 and 75 mRNAs in these categories (Figure 1).

### 3.2 Expression levels of islet GPCR ligand mRNAs in visceral fat from lean and obese mice

**FIGURE 2:**
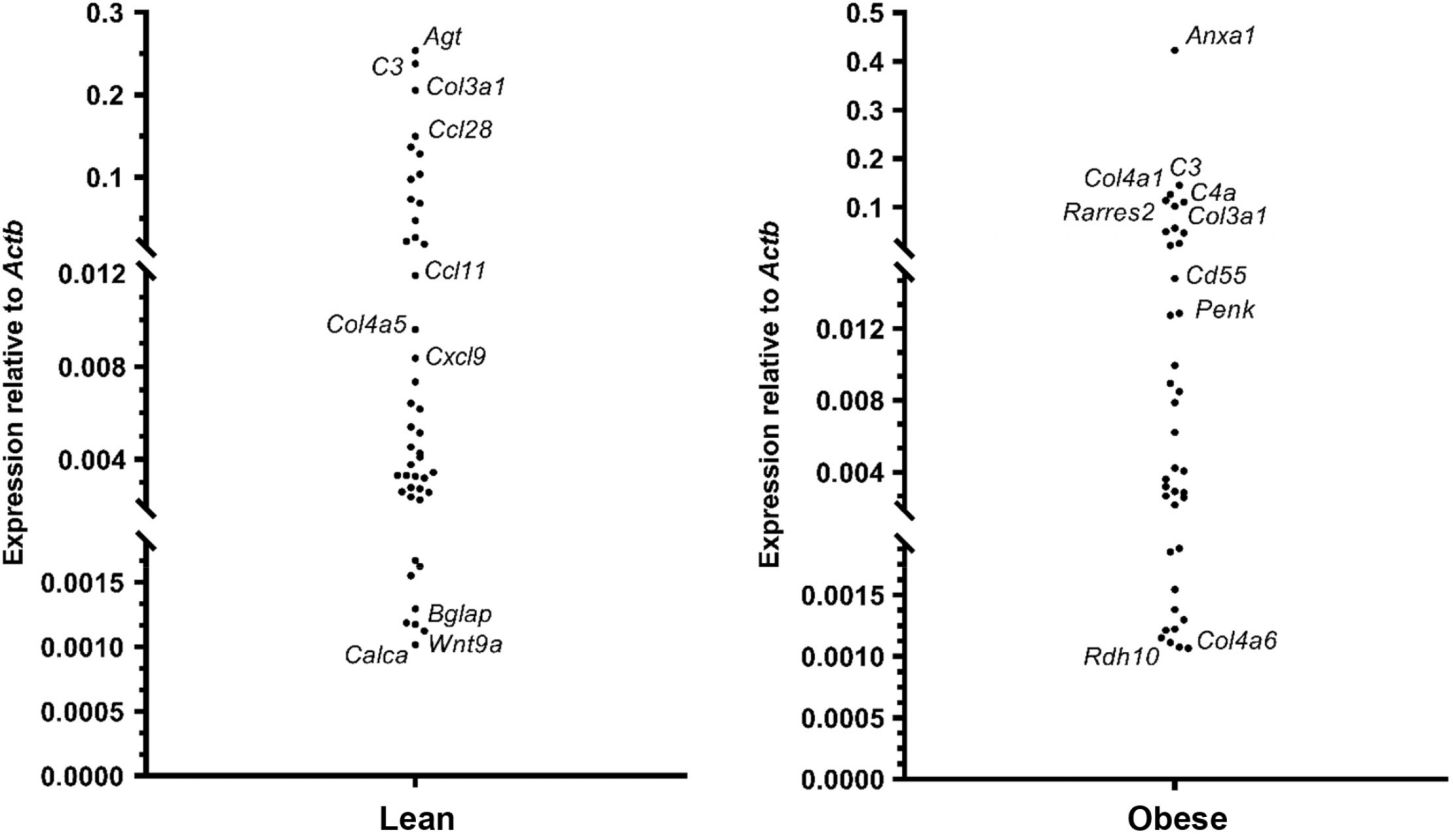
Islet GPCR ligand mRNA expression in visceral adipose tissue retrieved from lean (left) and obese (right) mice. Data were generated by RT‐qPCR and are displayed relative to expression of the housekeeping gene, Actb (*n* = 5 samples per group).

It can be seen from Figure 2 (left panel) that of the 45 mRNAs encoding islet GPCR ligands within visceral adipose tissue from lean mice, *Agt*, which codes for angiotensinogen, was the most highly expressed (0.25 ± 0.06 relative to *Actb*). There was also high expression of mRNAs coding for the complement peptide, *C3* (0.23 ± 0.07 relative to *Actb*), and for collagen type III α‐1 chain, *Col3a1* (0.20 ± 0.03 relative to *Actb*, Figure 2). In contrast, mRNAs encoding calcitonin peptide, *Calca* (0.0009 ± 0.0002 relative to *Actb*), Wnt signalling peptide, *Wnt9a* (0.001 ± 0.0002 relative to *Actb*), and bone protein, *Bglap* (0.001 ± 0.001 relative to *Actb*), were expressed at low levels in adipose tissue from lean mice (Figure 2, left panel). There was a different profile of islet GPCR ligand mRNA expression in visceral adipose tissue retrieved from obese mice, with *Anxa1*, which codes for annexin‐1, being the most abundant mRNA quantified (0.42 ± 0.07 relative to *Actb*; Figure 2, right panel). Some similarities in mRNA expression in adipose tissue from lean and obese mice were identified, with *C3* and *Col3a1* showing high expression in epididymal fat pads retrieved from mice fed standard chow and those fed a high‐fat diet fed (Figure 2).

### 3.3 Changes in islet GPCR ligand mRNA expression in visceral fat in obesity

**FIGURE 3:**
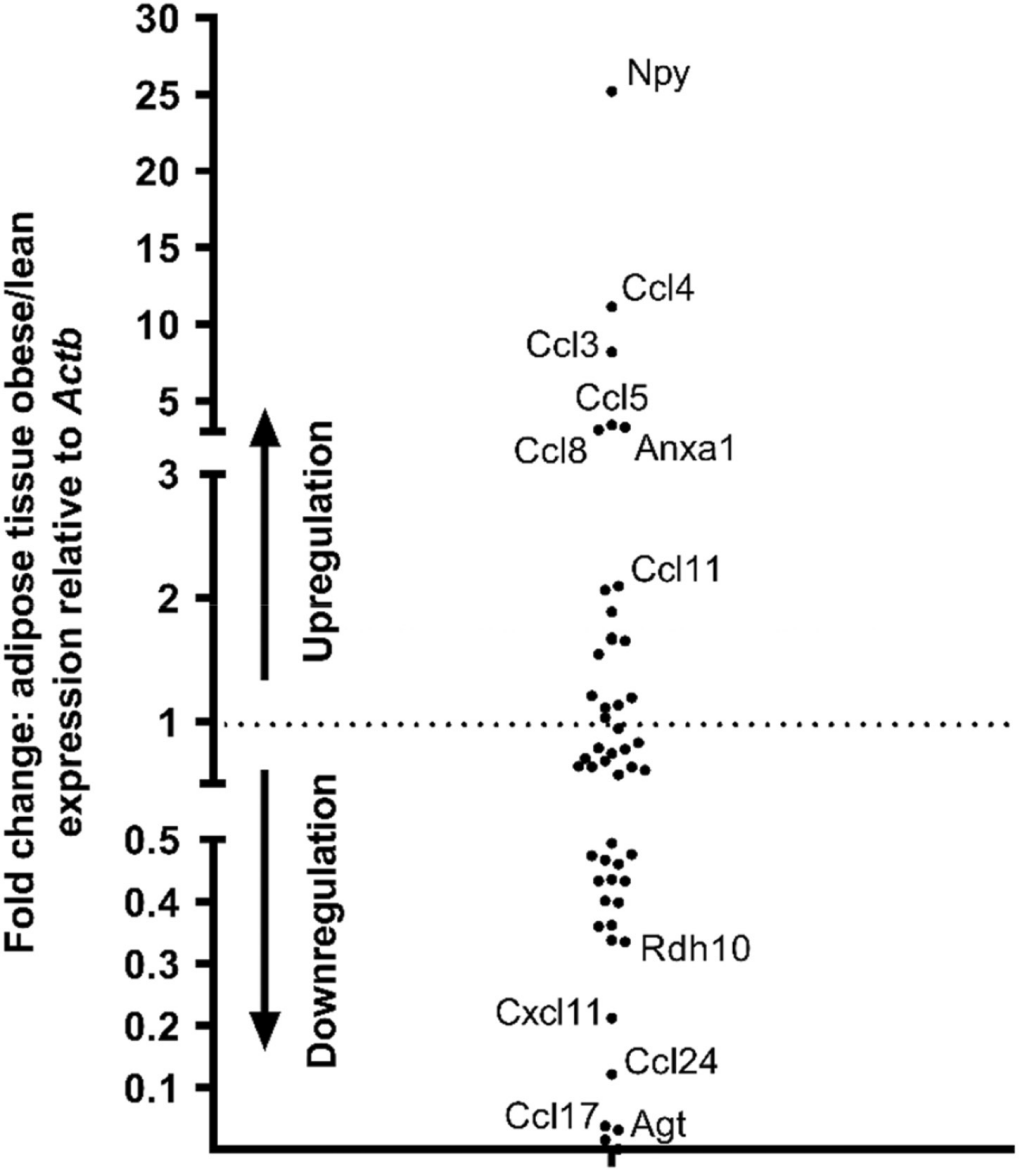
Up‐regulated and down‐regulated islet GPCR ligand mRNAs in visceral adipose tissue retrieved from lean and obese mice. Data were generated by RT‐qPCR and are displayed as fold change in mean expression values in obese versus lean mice, relative to the housekeeping gene, Actb (*n* = 5 samples per group).

It is clear from Figure 2 that visceral adipose tissue mRNA expression is not static and alterations occur after mice have been maintained on an obesogenic diet for 16 weeks. Figure 3 illustrates key islet GPCR‐targeting adipokine genes whose expression is either up‐regulated or down‐regulated in obesity, with a focus on those mRNAs whose expression was above trace level in adipose tissue from lean and/or obese mice. It can be seen that *Npy*, *Ccl4*, *Ccl3*, *Ccl5* and *Anxa1* mRNAs exhibit the most marked upregulation with high‐fat feeding (2502 ± 610%, 1112 ± 167%, 820 ± 200%, 342 ± 130% and 329 ± 54% of control diet expression respectively) and all of these, except *Ccl5*, showed significant upregulation when data from the five lean and five obese adipose tissue samples were compared (Table 1). Figure 3 also shows that mRNAs encoding *Agt* (96.9 ± 0.8% decrease), *Ccl17* (96.3 ± 0.8% decrease) and *Ccl24* (87.9 ± 0.5% decrease) were markedly down‐regulated with high‐fat feeding, although of these only changes in *Agt* were statistically significant (Table 1). It can also be seen from Table 1 that, as expected, *Lep* and *AdipoQ* were readily detectable in epididymal adipose tissue and there was a significant increase in *Lep* (286 ± 33% of control diet expression) and a decrease *in AdipoQ* (37.7 ± 7.3% of control diet expression) in obesity. Table 1 also indicates that some GPCR ligand mRNAs that were considered to be absent, because they were expressed at <0.0001% of *Actb* or expressed at trace levels in adipose tissue from lean mice, were significantly up‐regulated in obesity, while others were down‐regulated. Thus, *Ghrh*, *Cck*, *C5* and *Ccl2* were up‐regulated (1759 ± 618%, 1408 ± 314%, 1119 ± 370% and 955 ± 104% of control diet expression respectively) in epididymal adipose tissue isolated from obese mice, whereas there were significant reductions in several non‐abundant mRNAs including *Cxcl3* (94.7 ± 3.0% decrease) *Wnt4* (94.5 ± 1.3% decrease), *Wnt7b* (84.6 ± 3.4% decrease) and *Col4a4* (81.5 ± 5.0% decrease).

**TABLE 1:** Significantly up‐regulated and down‐regulated islet GPCR ligand mRNAs in visceral adipose tissue retrieved from control‐fat diet‐ and high‐fat diet‐fed mice

Gene	Expression level (% *Actb*)		Expression (% of control diet)	*p*‐value	Significance
	Control diet	High‐fat diet			
Significantly up‐regulated mRNAs					
*Npy*	0.000043	0.001076	2502%	0.0107	*
*Ccl4*	0.000169	0.001880	1112%	0.0004	***
*Ccl3*	0.000441	0.003614	820%	0.0076	**
*Anxa1*	0.128500	0.422500	329%	0.0048	**
*Ccl8*	0.003206	0.009947	310%	0.0114	*
*Ccl11*	0.011920	0.024970	209%	0.0403	*
*Ccl7*	0.006171	0.012730	206%	0.0342	*
*Wnt11*	0.000831	0.001380	166%	0.0295	*
Significantly down‐regulated mRNAs					
*Aldh1a2*	0.003311	0.001545	46.7%	0.0094	**
*Wnt9a*	0.001124	0.000517	46.0%	0.0316	*
*Edn1*	0.004273	0.001849	43.3%	0.0465	*
*Wnt2b*	0.005406	0.002169	40.1%	0.0119	*
*Rspo1*	0.001624	0.000647	39.8%	0.0488	*
*Calca*	0.001017	0.000368	36.2%	0.0248	*
*Rdh10*	0.003319	0.001111	33.5%	0.0207	*
*Agt*	0.253600	0.007879	3.1%	0.0042	**
Positive controls					
*Lep*	0.199300	0.569100	286%	0.0089	**
*AdipoQ*	1.272000	0.479400	37.7%	0.0759	NS
Significant changes in low abundance mRNAs					
*Ghrh*	0.000022	0.000387	1759%	0.0257	*
*Cck*	0.000050	0.000704	1408%	0.0033	**
*C5*	0.000016	0.000179	1119%	0.0259	*
*Ccl2*	0.000101	0.000965	955%	0.00004	****
*Ctsg*	0.000037	0.000183	496%	0.0042	**
*Xcl1*	0.000042	0.000071	169%	0.0090	**
*Wnt10b*	0.000291	0.000066	22.7%	0.0016	**
*Prok2*	0.000004	0.000001	25.0%	0.0322	*
*Col4a4*	0.000507	0.000094	18.5%	0.0125	*
*Wnt7b*	0.000052	0.000008	15.4%	0.0459	*
*Wnt4*	0.000862	0.000047	5.5%	0.0494	*
*Cxcl3*	0.000076	0.000004	5.3%	0.0039	**

*Note*: AdipoQ and Lep genes encode products that target non‐GPCR receptors and were included as adipose tissue positive controls. Data are expressed as % of control diet, relative to the housekeeping gene, Actb (*n* = 5 per group).

*p < 0.05.

**p < 0.01.

***p < 0.001.

****p < 0.0001.

## 4 DISCUSSION

Adipokines are secreted signalling peptides that have roles in a wide range of biological functions, including systemic lipid and glucose metabolism, local inflammation and adipocyte differentiation and metabolism. The degree of adiposity profoundly affects the level of expression and secretion of multiple adipokines. In a healthy individual, tight regulation exists to maintain a balance between the release of anti‐inflammatory, anti‐diabetic adipokines and pro‐inflammatory, pro‐diabetic adipokines. The expansion of adipose tissue stores in obesity, particularly visceral adipose tissue, leads to an imbalance of these opposing groups of peptides, such that the level of pro‐inflammatory and pro‐diabetic adipokines, such as TNF‐α and monocyte chemoattractant protein‐1 supersedes.^4^ It is unlikely that a single adipokine is responsible for the metabolic perturbations that accompany obesity, but multiple adipokines can operate cooperatively and/or synergistically to induce dysglycaemia. Not all adipokines are detrimental, however: adiponectin is an insulin‐sensitising peptide that also increases insulin output,^9^ and adipsin also improves glucose homeostasis through stimulation of insulin secretion.^10^ It is readily accepted that enteroendocrine cells of the gastrointestinal tract secrete incretin peptides that stimulate insulin secretion,^17^ but the concept of adipocyte‐islet crosstalk is not well‐established. One such incretin, glucagon‐like peptide 1 (GLP‐1), forms the basis of the only islet GPCR‐targeted therapy for T2D, and GLP‐1 receptor analogues such as exenatide and liraglutide have been used clinically for over a decade. Given that GPCRs are a large receptor family that represent ~35% of all current drug targets for treatment across a wide range of diseases,^18^ islets express mRNAs encoding nearly 300 GPCRs^14^ and adipocytes are a source of signalling peptides,^19^ we hypothesise that adipokine crosstalk with islets, via GPCRs, may be a mechanism through which functional β‐cell mass is regulated.

The aim of this study, therefore, was to profile mRNA expression of adipokines that have been identified as ligands for islet GPCRs in mouse visceral adipose tissue, and determine whether there were changes in expression in obesity. We found that <30% of these mRNAs were quantified at levels >0.001% of the housekeeping gene *Actb*, and all of these were expressed at considerably lower levels than the established adipokine, *AdipoQ*. The most abundant GPCR ligand mRNA in adipose tissue isolated from lean mice was *Agt*, but while islets express AT_1_ receptors and exogenous angiotensin stimulates insulin secretion,^20^ the systemic hypertensive effects of angiotensin preclude its use therapeutically in diabetes. In obese mouse adipose tissue *Anxa1*, which encodes the phospholipid‐binding protein annexin A1, was the most highly expressed ligand mRNA, and its expression increased in obesity to 329 ± 54% of the levels quantified in adipose tissue from lean mice. Consistent with our data, a recent high‐throughput screening study of alterations in mouse adipocyte transcriptional profiles identified that *Anxa1* expression in epididymal adipocytes isolated from mice maintained on a 60% fat diet for 12 weeks was 364 ± 26.4% of that quantified in adipocytes of mice fed a 10% fat diet.^21^ Annexin A1 is known to have beneficial effects in islets by potentiating glucose‐stimulated insulin release and protecting against apoptosis,^22^ so its secretion from expanded adipose depots in obesity may lead to improved islet function via binding to the formylpeptide receptor FPR2.

Recent transcriptomics analysis identified *Npy*, *Ccl4 and Ccl3* expression to be up‐regulated in visceral adipocytes of obese mice (1970 ± 89.1%, 417 ± 28.0% and 1250 ± 186.1% of expression levels in adipocytes from lean mice respectively; 21), which is broadly consistent with our data, shown in Table 1. If the peptides encoded by these up‐regulated mRNAs are secreted from adipocytes, they may regulate islet function via activation of Npy and chemokine receptors expressed by islets. While neuropeptide Y, encoded by *Npy*, has established effects on islet function, chemokine (C‐C motif) ligand 4 (Ccl4), encoded by *Ccl4*, has been identified here as a candidate for investigation of its effects on regulation of insulin secretion and functional β‐cell mass. A descriptive summary of Npy and Ccl4 can be found in Table 2, which provides an overview of their respective primary functions, target GPCRs and associated signalling pathways, level of expression and effects on β‐cell function and insulin sensitivity. Key information is provided below.

**TABLE 2:** Summary of the characteristics and functions of the most highly up‐regulated islet GPCR ligand mRNAs in mouse visceral adipose tissue with high‐fat feeding

	Npy	Ccl4
Primary function	Orexigenic neuropeptide^35^ regulating food intake and energy storage	Chemoattractant for immune cell migration^36^
Target GPCR(s)	Npy1r, Npy2r, Npy3r, Npy4r	Ccr1, Ccr2, Ccr5, Ccr9
Signalling pathway(s)	Gαi‐mediated inhibition of adenylate cyclase	Gαi‐mediated inhibition of adenylate cyclase
Expression level	Significant increase in T2D patient plasma^37^; down‐regulated Npy expression with β‐cell maturation^38^	Significant increase in T2D patient sample material (mixture of plasma, serum and blood)^31^; no differences between prediabetic patients and healthy controls^31^
Effect(s) on insulin secretion	Inhibits glucose‐stimulated insulin secretion^27^, ^39^, ^40^	N/A
Effect(s) on β‐cell mass	Promotes β‐cell proliferation^29^, ^41^ and protects against cytokine‐ and streptozotocin‐induced islet apoptosis^29^	Induces β‐cell inflammation via IL‐6 and TNF‐α expression^32^
Effect(s) on insulin sensitivity	Inhibits insulin‐stimulated glucose uptake in adipocytes by attenuation of GLUT4 translocation^26^	Anti‐CCL4 antibodies attenuate hyperglycaemia progression, improve insulin sensitvity and increase circulating insulin levels^32^

Abbreviation: N/A, not available.

We found that *Npy* was up‐regulated over 25‐fold in adipose tissue from obese mice and previous studies have indicated elevated plasma Npy levels in obese mice,^23^ and humans.^24^ Interestingly, the increase in circulating Npy in obese humans coincided with a pronounced upregulation in *Npy* expression within subcutaneous fat depots, suggestive of an adipose tissue source.^24^ The obese state is exacerbated further by associated increases in Npy levels, as Npy stimulates preadipocyte proliferation and white adipose tissue adipogenesis^23^ while inhibiting brown adipose tissue thermogenesis via down‐regulated uncoupling protein 1 expression.^25^ Moreover, delivery of exogenous Npy to lean and obese mice resulted in a 50% expansion of adipose tissue weight and volume.^23^ Treatment of 3 T3‐L1 adipoyctes with exogenous Npy inhibited insulin‐stimulated glucose uptake via diminished GLUT4 translocation, an effect that was abolished by a Y1‐specific receptor antagonist, suggesting involvement of this Npy target GPCR in glucose tolerance.^26^ Npy also inhibits glucose‐stimulated insulin secretion^27^ and islet‐specific Npy deletion in mice enhanced basal and glucose‐stimulated insulin secretion, which was attributed to a significantly greater islet area compared to their wild‐type littermates.^28^ However, despite its negative regulation of peripheral insulin sensitivity and insulin secretory function, there is evidence of Npy promoting β‐cell proliferation and protecting against cytokine‐ and streptozotocin‐induced islet apoptosis.^29^

Ccl4, also known as macrophage inflammatory protein‐1β, is a chemoattractant protein produced by endothelial and epithelial cells, fibroblasts, neutrophils, monocytes and lymphocytes, which has an established role in promoting leucocyte activation and recruitment to sites of inflammation.^30^ More recently, several studies have implicated Ccl4 in metabolic regulation. Thus, a meta‐analysis revealed that CCL4 levels are significantly higher in patients with T2D, while no differences were observed between healthy controls and patients with prediabetes, suggesting an association between CCL4 levels and diabetes progression.^31^ A functional role for Ccl4 in promoting dysglycaemia is suggested by observations that Ccl4 inhibition in *Lepr* ^*db*^/JNarl diabetic mice and high‐fat diet‐fed mice was associated with improved hepatic and muscle insulin sensitivity and delayed hyperglycaemia progression, and treatment of streptozotocin‐induced diabetic mice with anti‐Ccl4 antibodies increased islet cell proliferation and serum insulin levels, leading to improved plasma glucose control.^32^ These beneficial effects of Ccl4 blockade may be linked to reduction in Ccl4‐induced β‐cell inflammation as expression of the inflammatory cytokines, IL‐6 and TNF‐α, was reduced in the NIT‐1 β‐cell line by siRNA‐mediated knockdown of Ccr2 and Ccr5, GPCRs for which Ccl4 is a ligand.^32^ In support of these observations, another study which also utilised anti‐Ccl4 antibodies reported that Ccl4 inhibition improved insulin sensitivity and lipid profiles, delayed the progression of hyperglycaemia and reduced systemic inflammation in high‐fat diet‐fed mice.^33^ Ccl4 acts as a ligand for Ccr1, Ccr2, Ccr5 and Ccr9, and we have identified that mRNAs encoding CCR1 and CCR9 are significantly up‐regulated in human islets from obese subjects.^34^ There are no current reports on the role of Ccl4 on islet function, but the upregulation of mRNAs encoding adipose tissue Ccl4 and islet CCR1 and CCR9 in obesity suggests that increased release of Ccl4 from adipocytes in obesity could reduce insulin secretion via activation of one, or both, of these Gαi‐coupled islet receptors, exacerbating its effects to induce inflammation and insulin resistance. Under these circumstances, antagonists to CCR1 and/or CCR9 may be beneficial in improving β‐cell function and overall glucose homeostasis.

In summary, we have quantified mRNAs encoding islet GPCR ligands in adipose tissue from lean and obese mice and alterations in mRNA expression in obesity. The data generated point to ligands of interest for future research on adipose‐islet crosstalk via secreted ligands acting at islet GPCRs, and it will be important to determine whether the alterations in mRNA expression that we have identified here are accompanied by alterations in circulating levels of these ligands. Such research may identify islet GPCRs with therapeutic potential for T2D.

## CONFLICTS OF INTEREST

The authors have no conflicts of interest to declare.

## Supporting information


Table S1

## Data Availability

The data that support the findings of this study are available from the corresponding author upon reasonable request.
