# Supplementary material for: Alterations in mouse visceral adipose tissue mRNA expression of islet G‐protein‐coupled receptor ligands in obesity
Source: Diabet Med. 2022 Oct 26;39(12):e14978. doi: 10.1111/dme.14978 (PMC9828549; doi:10.1111/dme.14978)
Supplement: Supplementary file 1 — Table S1 [file DME-39-0-s001.docx]

**Supplementary**

| **Gene** | **Qiagen cat.** | **Amplicon** |
| --- | --- | --- |
| **(mouse)** | **number** | **length (bp)** |
| ***Adcyap1*** | QT00100317 | 92 |
| ***Adm*** | QT00249676 | 105 |
| ***Adm2*** | QT00269003 | 150 |
| ***Agt*** | QT00164745 | 61 |
| ***Aldh1a1*** | QT02330937 | 105 |
| ***Aldh1a2*** | QT00120477 | 86 |
| ***Aldh1a3*** | QT01077867 | 72 |
| ***Anxa1*** | QT00145915 | 170 |
| ***Apln*** | QT00111762 | 103 |
| ***Avp*** | QT00249389 | 93 |
| ***Bglap*** | QT00259406 | 102 |
| ***C1ql1*** | QT00259238 | 90 |
| ***C1ql2*** | QT00318227 | 126 |
| ***C1ql3*** | QT00282478 | 64 |
| ***C1ql4*** | QT01038520 | 100 |
| ***C3*** | QT00109270 | 108 |
| ***C4a*** | QT01074948 | 187 |
| ***Calca*** | QT01747865 | 96 |
| ***Calcb*** | QT00162078 | 94 |
| ***Cartpt*** | QT00130396 | 82 |
| ***Cck*** | QT00250145 | 91 |
| ***Ccl1*** | QT01044421 | 111 |
| ***Ccl11*** | QT00114275 | 109 |
| ***Ccl17*** | QT00131572 | 103 |
| ***Ccl19*** | QT02532173 | 68 |
| ***Ccl2*** | QT00167832 | 118 |
| ***Ccl20*** | QT02326394 | 71 |
| ***Ccl21a*** | QT00284753 | 96 |
| ***Ccl22*** | QT00108031 | 92 |
| ***Ccl24*** | QT00126021 | 116 |
| ***Ccl25*** | QT00110173 | 143 |
| ***Ccl26*** | QT01559481 | 99 |
| ***Ccl27a*** | QT00325003 | 190 |
| ***Ccl27b*** | QT02281895 | 113 |
| ***Ccl28*** | QT01752625 | 102 |
| ***Ccl3*** | QT00248199 | 107 |
| ***Ccl4*** | QT00154616 | 110 |
| ***Ccl5*** | QT01747165 | 107 |
| ***Ccl7*** | QT00171458 | 117 |
| ***Ccl8*** | QT00128548 | 87 |
| ***Cd55*** | QT00133994 | 121 |
| ***Cga*** | QT00098084 | 81 |
| ***Col3a1*** | QT02331301 | 73 |
| ***Cort*** | QT00104272 | 149 |
| ***Crh*** | QT01055789 | 183 |
| ***Ctsg*** | QT00112112 | 118 |
| ***Cx3cl1*** | QT00128345 | 98 |
| ***Cxcl1*** | QT00115647 | 93 |
| ***Cxcl10*** | QT00093436 | 92 |
| ***Cxcl11*** | QT00265041 | 86 |
| ***Cxcl12*** | QT00161112 | 71 |
| ***Cxcl13*** | QT00107919 | 64 |
| ***Cxcl16*** | QT00171535 | 129 |
| ***Cxcl2*** | QT00113253 | 81 |
| ***Cxcl3*** | QT00151599 | 133 |
| ***Cxcl4*** | QT00312529 | 82 |
| ***Cxcl5*** | QT01658146 | 62 |
| ***Cxcl9*** | QT00097062 | 98 |
| ***Edn1*** | QT00253512 | 116 |
| ***Edn2*** | QT00198814 | 77 |
| ***Edn3*** | QT00107905 | 140 |
| ***F2*** | QT00128870 | 137 |
| ***Fshb*** | QT00150262 | 109 |
| ***Gal*** | QT00109970 | 69 |
| ***Galp*** | QT02532796 | 121 |
| ***Gast*** | QT00107702 | 150 |
| ***Gcg*** | QT00124033 | 94 |
| ***Ghrh*** | QT00136787 | 121 |
| ***Ghrl*** | QT00137536 | 73 |
| ***Gip*** | QT00146692 | 140 |
| ***Gnhr1*** | QT01062600 | 124 |
| ***Grp*** | QT00101780 | 79 |
| ***Hcrt*** | QT00112252 | 156 |
| ***Iapp*** | QT00101052 | 73 |
| ***Insl3*** | QT01660855 | 189 |
| ***Insl5*** | QT00261345 | 146 |
| ***Kiss1*** | QT01764259 | 146 |
| ***Kng1*** | QT00106260 | 99 |
| ***Lhb*** | QT00262829 | 150 |
| ***Nmb*** | QT00105945 | 92 |
| ***Nms*** | QT01043672 | 127 |
| ***Nmu*** | QT00133091 | 100 |
| ***Npff*** | QT00319984 | 120 |
| ***Nps*** | QT02251396 | 69 |
| ***Npvf*** | QT01054340 | 118 |
| ***Npw*** | QT00295400 | 119 |
| ***Npy*** | QT00134302 | 150 |
| ***Nts*** | QT00127225 | 142 |
| ***Oxt*** | QT00252203 | 184 |
| ***Pdny*** | QT00110117 | 143 |
| ***Penk*** | QT00240961 | 95 |
| ***Pmch*** | QT01060339 | 106 |
| ***Pnoc*** | QT00102480 | 85 |
| ***Pomc*** | QT00162218 | 99 |
| ***Ppbp*** | QT00160993 | 96 |
| ***Ppy*** | QT00103999 | 150 |
| ***Prlh*** | QT01564941 | 99 |
| ***Prok1*** | QT02326296 | 184 |
| ***Prok2*** | QT00133910 | 62 |
| ***Prss1*** | QT01757651 | 113 |
| ***Pth*** | QT00161539 | 96 |
| ***Pth2*** | QT00284102 | 80 |
| ***Pthlh*** | QT00114982 | 119 |
| ***Pyy*** | QT00123277 | 79 |
| ***Qrfp*** | QT00136395 | 83 |
| ***Rarres2*** | QT00117166 | 84 |
| ***Rdh10*** | QT00159824 | 106 |
| ***Rln1*** | QT01071112 | 73 |
| ***Rln3*** | QT01073996 | 97 |
| ***Rspo1*** | QT00124831 | 67 |
| ***Rspo2*** | QT00154182 | 129 |
| ***Rspo3*** | QT00127988 | 138 |
| ***Rspo4*** | QT00136234 | 119 |
| ***Sct*** | QT00249361 | 134 |
| ***Sst*** | QT01046528 | 89 |
| ***Tac1*** | QT01037141 | 154 |
| ***Trh*** | QT01745023 | 109 |
| ***Tshb*** | QT00135303 | 95 |
| ***Ucn*** | QT00326879 | 112 |
| ***Ucn2*** | QT01556534 | 125 |
| ***Ucn3*** | QT00302267 | 125 |
| ***Uts2*** | QT00124278 | 92 |
| ***Vip*** | QT00133966 | 96 |
| ***Wnt1*** | QT00103985 | 86 |
| ***Wnt2*** | QT00118503 | 119 |
| ***Wnt3*** | QT00133686 | 71 |
| ***Wnt3a*** | QT00250439 | 94 |
| ***Wnt4*** | QT00104622 | 134 |
| ***Wnt5a*** | QT00164500 | 130 |
| ***Wnt7b*** | QT00168812 | 114 |
| ***Xcl1*** | QT00095228 | 69 |
| ***Col4a1*** | QT00100128 | 92 |
| ***Col4a2*** | QT00141897 | 114 |
| ***Col4a3*** | QT01055537 | 63 |
| ***Col4a4*** | QT01055530 | 64 |
| ***Col4a5*** | QT01055467 | 150 |
| ***Col4a6*** | QT01041026 | 103 |
| ***Ndp*** | QT00105497 | 75 |
| ***Thy1*** | QT00245287 | 84 |
| ***Wnt10a*** | QT00110089 | 130 |
| ***Wnt10b*** | QT00113211 | 134 |
| ***Wnt11*** | QT00103663 | 106 |
| ***Wnt16*** | QT00134904 | 94 |
| ***Wnt2b*** | QT00115451 | 98 |
| ***Wnt5b*** | QT00169708 | 88 |
| ***Wnt6*** | QT01660883 | 111 |
| ***Wnt7a*** | QT00131719 | 99 |
| ***Wnt8a*** | QT00098035 | 132 |
| ***Wnt8b*** | QT02262470 | 104 |
| ***Wnt9a*** | QT01062250 | 139 |
| ***Wnt9b*** | QT00144256 | 101 |
| ***C5*** | QT00102032 | 90 |
| ***Tac2*** | QT02246734 | 74 |
| ***Cxcl17*** | QT01066058 | 142 |
| ***Cxcl14*** | QT00171157 | 76 |
| **Control** | **Qiagen cat.** | **Amplicon** |
| **Gene** | **number** | **length (bp)** |
| ***AdipoQ*** | QT01048047 | 135 |
| ***Lep*** | QT00164360 | 134 |

**Supplementary Table 1. List of QuantiTect primers (Qiagen Ltd.) used for qPCR in this study.**
